# Data on strategically located land and spatially integrated urban human settlements in South Africa

**DOI:** 10.1016/j.dib.2017.10.044

**Published:** 2017-10-28

**Authors:** Walter Musakwa

**Affiliations:** Department of Town and Regional Planning, University of Johannesburg, Johannesburg, South Africa

**Keywords:** Strategically located land, Spatial, land, Integrated human settlements

## Abstract

In developing countries like South Africa processed geographic information systems (GIS) data on land suitability, is often not available for land use management. Data in this article is based on a published article “The strategically located land index support system for humans settlements land reform in South Africa” (Musakwa et al., 2017) [1]. This article utilities data from Musakwa et al. (2017) [1] and it goes on a step further by presenting the top 25th percentile of areas in the country that are strategically located and suited to develop spatially integrated human settlements. Furthermore the least 25th percentile of the country that are not strategically located and spatially integrated to establish human settlements are also presented. The article also presents the processed spatial datasets that where used to develop the strategically located land index as supplementary material. The data presented is meant to stir debate on spatially integrated human settlements in South Africa.

**Specifications Table**

**Table:** 

Subject area	Urban and Regional Planning
More specific subject area	Land suitability analysis
Type of data	Geographic Information Systems (GIS) data
How data was acquired	Spatial data relating to strategically located land was collected from various government departments.
Data format	Analysed
Experimental factors	Spatial criteria relating to strategic location of land for human settlements was identified from literature and a consultative workshop was conducted to reach a consensus on selecting 15 criterion used to identify strategically located land and spatially integrated land for human settlements [1]. The criterion was weighted to establish the relative importance of each criterion in developing the strategically located index using the group analytical hierarchy calculator developed by [2].
Experimental features	The weighted linear combination (WLC) tool in ArcGIS software was used to compute the strategically located land index (SLLI) for human settlements in South Africa [1]
Data source location	South Africa.
Data accessibility	Data is with this article

**Value of the data**

• This data is useful because, it maps out well-located land in South Africa to establish smart human settlements.
• The data can be used to facilitate decision making for human settlements land reform, and other land use management needs.
• The maps and the data are useful for other researchers, urban planners and policy makers.
• The maps provide a visual picture of strategically located land in South Africa.
• The data is useful in the on-going debate on how successful has the new democratic dispensation in promoting spatial integration.

## 1 Data

**Fig. 1:**
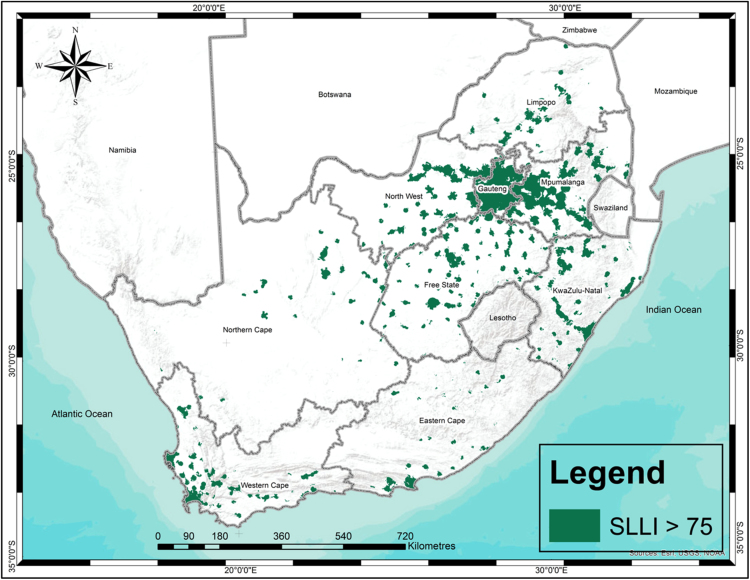
Highly strategic land or land with a strategically located land of over 75.

The SLLI ranges from 0 to 100 where a value close to zero implies unstrategically located land to establish human settlements whereas a value close to 100 implies highly strategic land to establish human settlements. Fig. 1 goes a step further from [1] as it visualises the top 25th percentile of strategically located land (SLLI<75) that contains the necessary services, infrastructure and amenities to establish integrated human settlements.

**Fig. 2:**
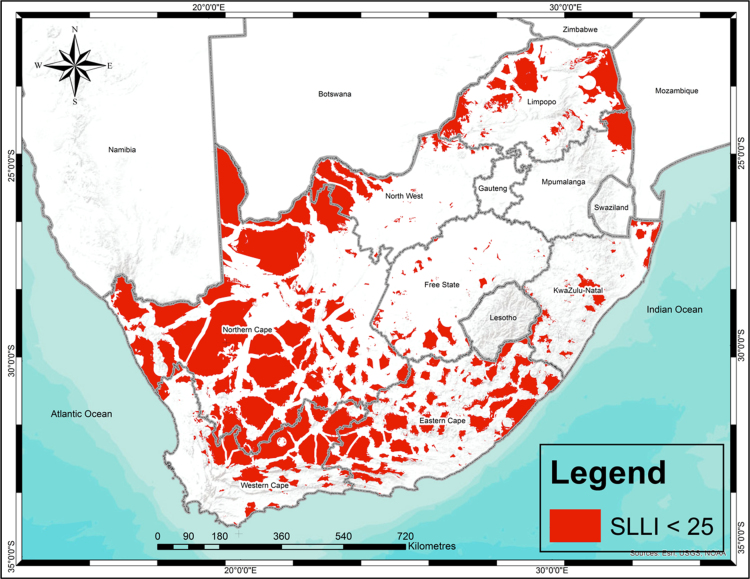
Highly unsuitable land with a strategically located land index of less than 25.

Similarly, Fig. 2 shows the lowest 25th percentile (SLLI>25) of land in South Africa that is not strategically located and contains minimal services, infrastructure and amenities to support integrated human settlements.

## 2 Materials and methods

The strategically located land index was developed using geographic information system and multi-criteria decision analysis (GIS-MCDA) techniques. Fourteen spatial criteria and datasets relating to human settlements underwent a GIS-MCDA process as described in [1] to develop the strategically located land index for human settlements. The query builder function of ArcGIS 10.3 was then used to extract the highly strategically located land and the unstrategically located land.
